# Immunogenicity and preclinical efficacy characterization of ShecVax, a combined vaccine against *Shigella* and enterotoxigenic *Escherichia coli*

**DOI:** 10.1128/iai.00004-25

**Published:** 2025-04-10

**Authors:** Siqi Li, Ipshita Upadhyay, Hyesuk Seo, Sai S. R. Vakamalla, Aashwina Madhwal, David A. Sack, Weiping Zhang

**Affiliations:** 1 Department of Pathobiology, University of Illinois at Urbana—Champaign 14589, Urbana, Illinois, USA; 2 Department of Pathobiology and Diagnostic Medicine, Kansas State University 5308, Manhattan, Kansas, USA; 3 Department of International Health, Johns Hopkins University Bloomberg School of Public Health 25802, Baltimore, Maryland, USA; University of California San Diego School of Medicine, La Jolla, California, USA

**Keywords:** *Shigella*, enterotoxigenic *Escherichia coli *(ETEC), combined vaccine, vaccinology platform, multiepitope fusion antigen (MEFA), diarrhea, EPI

## Abstract

No licensed vaccines are available for the largely antibiotic-resistant *Shigella* or enterotoxigenic *Escherichia coli* (ETEC), the two most common bacteria causing children’s diarrhea and travelers’ diarrhea. Virulence heterogeneity is a key obstacle to developing vaccines against *Shigella* or ETEC. By applying a multiepitope fusion antigen (MEFA) vaccinology platform, we recently constructed epitope- and structure-based polyvalent proteins to induce cross-protective antibodies against heterogeneous *Shigella* or ETEC strains. In this study, we combined a polyvalent *Shigella* protein with two polyvalent ETEC proteins, examined antigen compatibility and broad immunogenicity, and evaluated the potential of developing a combined vaccine against the two groups of bacteria. Data showed that mice intramuscularly immunized with the combined vaccine candidate (ShecVax) developed antibodies to all the following target virulence factors: *Shigella* IpaB, IpaD, VirG, GuaB, StxA, Stx2A, and StxB, and ETEC STa, LT, CFA/I, CS1, CS2, CS3, CS4, CS5, and CS6. ShecVax-induced antibodies significantly inhibited the invasion of all *Shigella* species and important serotypes, prevented the adherence of all important ETEC pathotypes, and neutralized the enterotoxicity of ETEC toxins STa and LT. Moreover, ShecVax prevented mice from lethal pulmonary infection with *Shigella sonnei* or *S. flexneri* 2a, significantly reduced ETEC bacterial colonization in rabbit small intestines, and passively protected newborn pigs against ETEC toxin-mediated clinical diarrhea. These results indicated that ShecVax is broadly immunogenic and cross-protective against *Shigella* and ETEC, suggesting ShecVax can be a *Shigella*/ETEC combined vaccine against children’s and travelers’ diarrhea, and the MEFA platform can be generally applied for vaccine development against heterogeneous pathogens or different diseases.

**IMPORTANCE:**

There are no effective countermeasures against *Shigella* and enterotoxigenic *E. coli* (ETEC), two antibiotic-resistant groups of bacteria and the leading causes of diarrhea in children in developing countries (children’s diarrhea) and international travelers (travelers’ diarrhea). Vaccines are a more practical approach to protect against infectious diseases, including diarrhea caused by *Shigella* or ETEC. A combined vaccine cross-protective against *Shigella* and ETEC can save hundreds of thousands of lives and prevent hundreds of millions of diarrhea cases yearly; it can also reduce antibiotic prescription and decrease antibiotic resistance, thus significantly improving global health. In addition, we may apply the MEFA platform to develop combined vaccines against heterogeneous pathogens or different diseases to accommodate an increasingly crowded expanded program on immunization (EPI).

## INTRODUCTION

Diarrheal disease causes nearly one million deaths annually and remains a significant burden for global health (1). *Shigella* spp. and enterotoxigenic *Escherichia coli* (ETEC) are the most common bacteria that cause diarrhea in children and adults in low- and middle-income countries (LMIC) and international travelers (1, 2), with an estimated 270 million cases and 212,000 deaths due to *Shigella* and 220 million cases and 51,000 deaths due to ETEC (1). While diarrheal mortality has decreased in recent years because of global socioeconomic improvement and healthcare advocacy (including oral rehydration solutions and vaccines), *Shigella* and ETEC long-term morbidity has persisted for decades (3, 4).

Effective countermeasures against enteric infections would improve global health and reduce the prescription of antibiotic drugs and the impact of antimicrobial resistance (AMR) (3–8). Rapid improvements in water–sanitation–hygiene (WASH) are unlikely to be achieved in most LMICs; concurrently, treatment with antibiotics is becoming less effective due to increasing AMR in *Shigella* and ETEC bacteria (9–16). Vaccines constitute a viable solution to protect against shigellosis and ETEC diarrhea. Despite being a high priority for the World Health Organization (WHO), United Nations Children’s Fund (UNICEF), and many other public health institutes (8, 17), a licensed vaccine for *Shigella* or ETEC has not been achieved.

There are challenges in developing vaccines for ETEC and *Shigella*. ETEC or *Shigella* bacteria are considerably heterogeneous. *Shigella* has four species (*S. flexneri*, *S. sonnei*, *S. boydii*, and *S. dysenteriae*) and over 50 serotypes. ETEC pathotypes produce more than 25 immunologically different adhesins (colonization factor antigens [CFAs] and coli surface antigens [CSs]) to attach host receptors and colonize the small intestine. ETEC strains also produce two distinctive enterotoxins, heat-labile toxin (LT) and heat-stable toxin (STa), to stimulate fluid hyper-secretion, which causes watery diarrhea. Another challenge is to protect young children in LMICs who respond less well to oral vaccines (18, 19), while an oral vaccine for ETEC or *Shigella* must be designed to protect infants <1 year. Additionally, with the continuous introduction of new vaccines, the vaccine schedule for infants and young children is getting very crowded, so additional vaccines will need to fit with the current Expanded Program on Immunization (EPI) strategy.

Overcoming these challenges will be crucial for developing an effective *Shigella* or ETEC vaccine. An ideal ETEC vaccine would prevent all ETEC pathotypes from colonizing host small intestines and neutralize the enterotoxicity of both ETEC toxins (3, 20–23); an optimal *Shigella* vaccine would protect against all *Shigella* spp. and serotypes (4, 24, 25). While such vaccines are currently not achievable, developing a vaccine against the most prevalent and virulent ETEC pathotypes or the *Shigella* serotypes associated with most moderate to severe diarrheal cases may be feasible (4, 22). ETEC pathotypes producing seven adhesins (CFA/I, CS1-CS6) and enterotoxin STa and/or LT are the most important for children’s diarrhea and travelers’ diarrhea (26–28). *Shigella sonnei* and *S. flexneri* cause 90% of moderate-to-severe shigellosis cases (29), with serotypes *S. sonnei*, *S. flexneri* 2a, 3a, and 6 responsible for the majority of these illnesses (24, 29, 30).

To overcome virulence heterogeneity and develop cross-protective vaccines against the divergent ETEC pathotypes or *Shigella* spp. and serotypes, we developed an epitope- and structure-based vaccinology platform termed multiepitope fusion antigen (MEFA) and applied this technology to construct broadly immunogenic and cross-protective polyvalent protein immunogens (31). This MEFA vaccinology platform integrates multiple foreign epitopes, including B-cell (or T-cell) immunodominant epitopes predicted *in silico* or functional epitopes identified empirically, into a structurally stable and immunogenic backbone immunogen, thus creating a polyvalent protein immunogen for broad immunity and cross-protection (31). By using *Shigella* invasion plasmid antigen D (IpaD) as a backbone and integrating *in silico* predicted immunodominant and conserved B-cell epitopes from IpaB, VirG (IcsA), GuaB, and Shiga toxins into the IpaD backbone, we constructed a MEFA-based polyvalent protein for *Shigella* spp. and serotypes, *Shigella* MEFA. Since IpaB, IpaD, VirG (IcsA), and GuaB are the virulence determinants of shigellosis and shared across *Shigella* spp. and serotypes, and Shiga toxins are the virulence determinant of dysentery for *S. dysenteriae* type 1 and Shiga toxin-producing *E. coli* (STEC), a polyvalent immunogen carrying antigenic elements (epitopes) of these virulence determinants can be broadly immunogenic and cross-protective. Indeed, the resultant *Shigella* MEFA protein induced antibodies that inhibited invasion from all *Shigella* species and important serotypes and neutralized cytotoxicity of Shiga toxins. With this *Shigella* MEFA protein as the antigen, we developed a vaccine candidate (ShigVax) to protect infections from the important *Shigella* spp. and serotypes (32).

Similarly, assisted with the MEFA platform, we constructed a polyvalent protein from ETEC adhesins, CFA/I/II/IV MEFA, to target the seven most important ETEC adhesins (CFA/I, CS1-CS6). These seven ETEC adhesins are associated with two-thirds of ETEC diarrheal cases. We also created a toxoid fusion protein, 3xSTa_N12S_–mnLT_R192G/L211A_, to cover both ETEC enterotoxins (LT, STa). LT and STa, alone or together, are produced by all ETEC pathotypes and associated with all ETEC diarrheal cases. We further demonstrated that CFA/I/II/IV MEFA protein induced cross-protective antibodies that protected against adherence and intestinal colonization from ETEC pathotypes expressing any of the seven adhesins targeted (33–35). We also showed that the toxoid fusion protein induced antibodies that neutralized both toxins and protected against ETEC toxin-mediated clinical diarrhea (36, 37).

In this study, we combined three MEFA-assisted polyvalent proteins (*Shigella* MEFA, ETEC CFA/I/II/IV MEFA, and ETEC toxoid fusion) as the antigens for a *Shigella* and ETEC combined vaccine candidate, examined antigenic compatibility of three proteins, and evaluated vaccine broad immunogenicity and efficacy against *Shigella* and ETEC infections in animal models. We also assessed the general utility of the MEFA platform in developing combined vaccines against different diseases and the potential solution of using combined vaccines to solve the challenge of an increasingly crowded EPI.

## RESULTS

### MEFA-assisted *Shigella* and ETEC combined vaccine candidate ShecVax is broadly immunogenic

**Fig 1:**
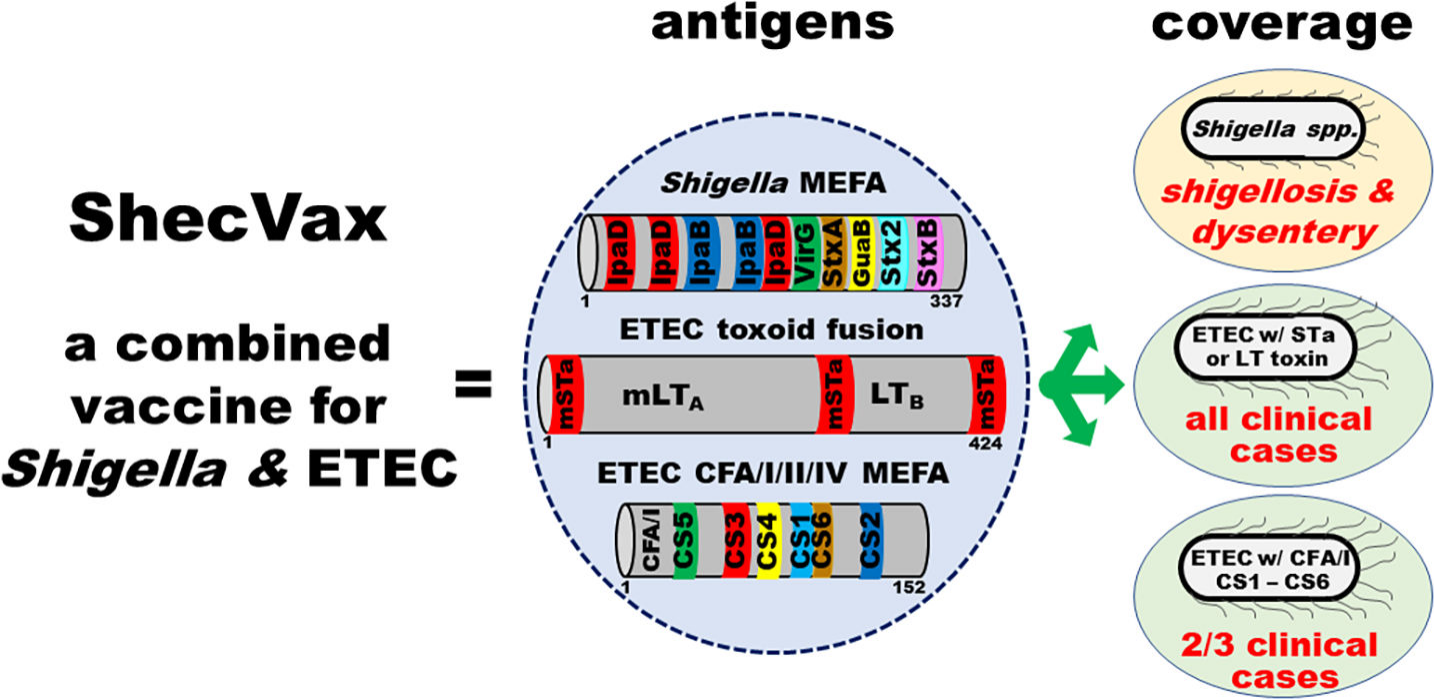
*Shigella* and ETEC combined vaccine candidate ShecVax antigen composition and protection coverage. ShecVax is composed of three polyvalent proteins (*Shigella* MEFA, ETEC toxoid fusion, ETEC CFA/I/II/IV MEFA) that were generated with the MEFA vaccinology platform. *Shigella* MEFA uses IpaD as the backbone immunogen to present B-cell immunodominant epitopes of *Shigella* virulence factors IpaB, IpaD, VirG, GuaB, and the A and B subunits of Shiga toxins Stx and Stx2. ETEC toxoid fusion 3xSTa_N12S_–mnLT_R192G/L211A_ has three STa toxoid STa_N12S_ genetically fused to a mutant LT monomer (an LT A subunit mutant and an LT B subunit) to target both ETEC enterotoxins (STa, LT). ETEC CFA/I/II/IV MEFA uses adhesin CFA/I subunit CfaB as the backbone to present B-cell immunodominant epitopes of the major subunits of the seven most important ETEC adhesins (CFA/I, CS1–CS6). ShecVax is for protecting against all shigellosis, dysentery, and ETEC-induced diarrhea cases.

The MEFA platform combines epitope vaccinology and structure vaccinology concepts. This platform assists in the construction of a polyvalent protein immunogen by substituting backbone protein epitopes with foreign epitopes from heterogeneous virulence determinants or virulent strains and further mimicking foreign epitope native antigenic properties (31). The resultant polyvalent MEFA immunogen can induce broad immunity to all target virulence factors or strains and serve as the antigen for a cross-protective multivalent vaccine. Assisted with this MEFA platform, we created polyvalent protein antigens, *Shigella* MEFA, ETEC toxoid fusion 3xSTa_N12S_–mnLT_R192G/L211A_, and ETEC CFA/I/II/IV MEFA. The *Shigella* MEFA uses IpaD as the backbone and carries immunodominant and conserved (across *Shigella* spp. and serotypes) B-cell epitopes of IpaB, IpaD, VirG, GuaB, and the A and B subunits of Shiga toxins (32). The ETEC toxoid fusion protein 3xSTa_N12S_–mnLT_R192G/L211A_ is a genetic fusion of three STa toxoids STa_N12S_ and monomeric LT mutant mnLT_R192G/L211A_ (38). STa_N12S_ has the 12th asparagine replaced with serine. The monomeric LT mutant has a mutant LT A subunit peptide, which has the 192nd arginine and the 211th leucine replaced, respectively, by glycine and alanine, fused to one LT B subunit for an A_1_B_1_ polypeptide. The CFA/I/II/IV MEFA is a polyvalent protein using adhesin CFA/I major subunit as the backbone to present epitopes of the seven most important ETEC adhesins (CFA/I, CS1, CS2, CS3, CS4, CS5, and CS6) (33). By combining these three polyvalent antigens, we constructed a *Shigella* and ETEC combined vaccine candidate, ShecVax, to protect against all cases of shigellosis, dysentery (caused by *Shigella* and Shiga toxin-producing *E. coli*, another major threat to public health), and ETEC diarrhea (Fig. 1).

**Fig 2:**
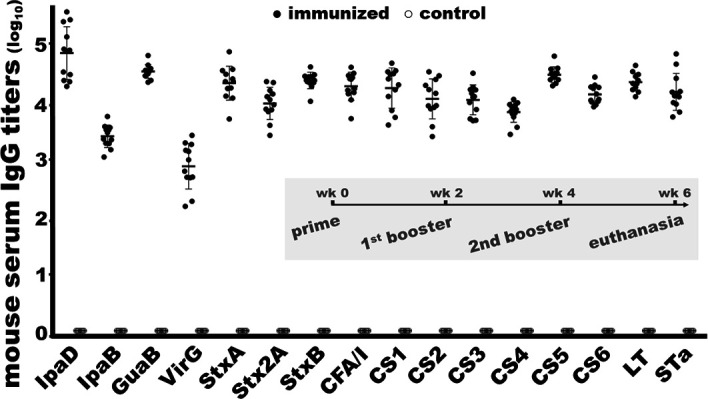
Antigen-specific IgG titers (log_10_) from the sera of the mice i.m. immunized with ShecVax or PBS (*n* = 12). Sera collected 2 weeks after the second booster from each mouse in the group immunized with ShecVax (●) or PBS (○) were titrated in ELISAs, with recombinant protein IpaD, IpaB, GuaB, VirG, Shiga toxin A subunit (StxA), Shiga toxin 2A subunit (Stx2A), Shiga toxin B subunit (StxB), CFA/I major subunit CfaB, CS1 subunit CooA, CS2 subunit CotA, CS3 subunit CstH, CS4 subunit CsaB, CS5 subunit CsfA, or CS6 subunit CssA, cholera toxin (CT; Sigma), or STa–ovalbumin conjugates as the coating antigen. HRP-conjugated goat-anti-mouse IgG (1:5,000; Bethyl Laboratories, Montgomery, TX, USA) is the secondary antibody. After subtracting from the background reading, the highest dilution that gave an OD value greater than 0.3 was calculated to IgG titers and converted to log_10_. A mouse i.m. immunization schedule scheme was included.

Mice intramuscularly (i.m.) immunized with ShecVax developed IgG antibodies to all target antigens (Fig. 2). The IgG titers (log_10_) to IpaD, IpaB, GuaB, VirG, StxA, Stx2A, StxB, CFA/I, CS1, CS2, CS3, CS4, CS5, CS6, LT, or STa from sera of the immunized mice, which were collected 2 weeks after the second booster, were 4.9 ± 0.45, 3.5 ± 0.19, 4.6 ± 0.12, 2.9 ± 0.39, 4.4 ± 0.29, 4.0 ± 0.28, 4.4 ± 0.14, 4.3 ± 0.24, 4.3 ± 0.35, 4.1 ± 0.35, 4.1 ± 0.26, 3.9 ± 0.18, 4.5 ± 0.13, 4.2 ± 0.16, 4.4 ± 0.16, and 4.2 ± 0.32, respectively.

No IgG responses to the target antigens were detected from the control mouse sera or sera collected before the primary immunization. No antigen-specific IgA antibodies were detected from the immunized or control mouse serum samples.

### ShecVax-induced antibodies are broadly functional against Shigella bacterial invasion, ETEC bacterial adherence, and ETEC toxin enterotoxicity

**Fig 3:**
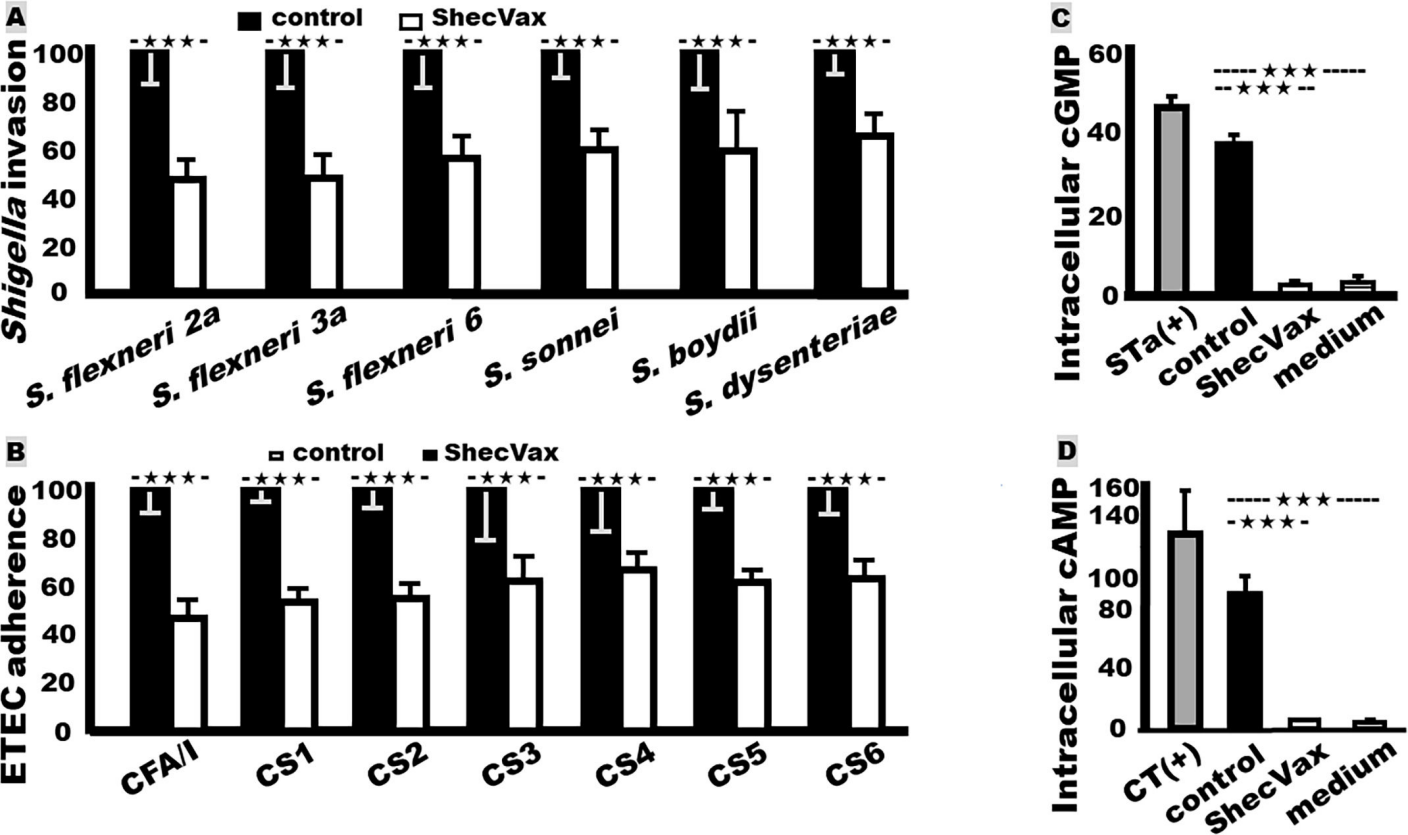
Mouse serum antibody functions (*in vitro*) against *Shigella* bacterial invasion, ETEC bacterial adherence, and ETEC STa and LT toxin enterotoxicity. (A) Sera from the group i.m. immunized with ShecVax (white box) or PBS (black box) were incubated with *S. flexneri* 2a, 3a, 6, *S. sonnei*, *S. boydii*, or *S. dysenteriae* bacteria, and then transferred to HeLa cells. Invaded *Shigella* bacteria (CFUs; in %) were collected, cultured, counted, and expressed in percentage by referring to the CFUs of bacteria treated with the control sera (as 100%). (B) Sera from the group i.m. immunized with ShecVax (white box) or PBS (black box) were incubated with ETEC field isolate H10407 (CFA/I), EL392-75 (CS1), ETP05011 (CS2), E116 (CS3), E106 (CS4), UM75688 (CS5), or B7A (CS6), then transferred to Caco-2 cells. Adherent bacteria (CFUs; in %) were collected, cultured, counted, and referred to the CFUs of bacteria treated with the control sera (100%). (C) Serum antibody neutralization against ETEC STa enterotoxicity using T-84 cells (ATCC) and an EIA cyclic GMP kit (Enzo Life Sciences). (D) Serum antibody neutralization against ETEC LT enterotoxicity using T-84 cells and an EIA cAMP kit. Mouse sera from the group i.m. immunized with ShecVax (white box) or PBS (black box) were incubated with toxin STa (2 ng) or cholera toxin (CT, 10 ng) and then transferred to T-84 cells. Intracellular cGMP or cAMP levels (nM; Pico moles per mL) were calculated by following the manufacturer’s protocol. T-84 cells treated with STa or CT without mouse sera (gray box) were used as a positive control, and cells cultured in medium (without toxin or mouse sera; box with horizontal bars) showed baseline cGMP or cAMP levels. Boxes and bars indicate means and deviations, and ***P < 0.0001.

*ShecVax-induced antibodies inhibited Shigella spp. and serotype invasion in vitro*. Mouse sera from the group i.m. immunized with combined vaccine candidate ShecVax inhibited invasion of *S. flexneri* 2a, 3a, 6, *S. sonnei*, *S. boydii*, and *S. dysenteriae* (Fig. 3A). After incubation with sera of the immunized mice, *S. flexneri* 2a, 3a, 6, *S. sonnei*, *S. boydii*, and *S. dysenteriae* invasion to Hela cells was reduced by 52%, 51%, 43%, 40%, 40%, and 34%, respectively, compared to invasion by the same bacteria treated with the control mouse sera.

*ShecVax-induced antibodies inhibited adherence of ETEC bacteria expressing CFA/I, CS1-CS6 adhesins in vitro*. Incubated with the sera of the mice immunized with ShecVax, adherence of ETEC H10407 (CFA/I, STa, LT), EL392-75 (CS1, CS3, STa, LT), ETP05011 (CS2, CS3, STa, LT), E116 (CS3, STa, LT), E106 (CS4, CS6, STa, LT), UM75688 (CS5, CS6, STa, LT), and B7A (CS6, STa, LT) to Caco-2 cells was reduced by 53%, 46%, 44%, 37%, 33%, 38%, and 36% respectively, significantly lower than the bacteria incubated with the control mouse sera (Fig. 3B).

*ShecVax-induced antibodies neutralized the enterotoxicity of two ETEC toxins (STa and LT*). Sera from the mice i.m. immunized with ShecVax neutralized STa and CT (cholera toxin, a structural and functional homolog of LT) enterotoxicity, shown by prevention of the toxin from elevating intracellular cyclic GMP (Fig. 3C) or AMP (Fig. 3D) levels in T-84 cells. Intracellular cGMP levels in the T-84 cells incubated with toxin STa treated with sera of the immunized mice were 3.7 ± 0.33 nM (pico moles per mL), significantly lower than the intracellular cGMP in the cells incubated with STa treated with the control mouse sera (38 ± 3.1 nM; *P* < 0.001), but not different from the baseline intracellular cGMP levels (4.5 ± 2.0 nM).

The intracellular cAMP levels in the T-84 cells incubated with CT treated with sera of the immunized mice were 5.0 ± 0.62 nM, significantly lower than the cAMP in the cells incubated with CT treated with the control mouse sera (88.5 ± 16.8 nM; *P* < 0.001), but not different from the T-84 cell baseline cAMP (4.8 ± 0.75 nM).

### ShecVax polyvalent proteins are antigenically compatible for co-administration

**Fig 4:**
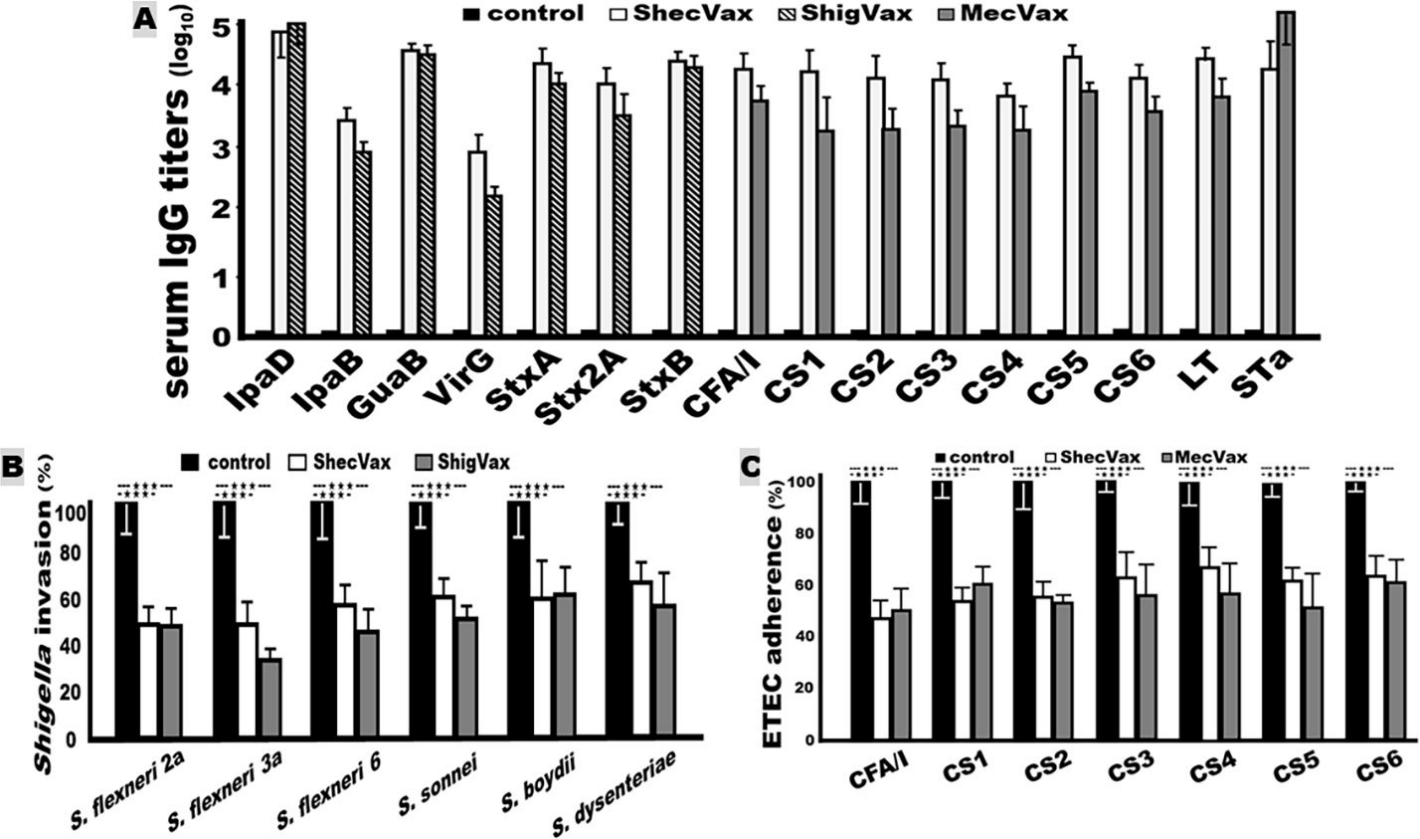
ShecVax immunogen antigenic compatibility assessment. (A) IgG titers (log_10_) from the sera of the mice i.m. immunized with ShecVax (white box; three protein antigens, *Shigella* MEFA, ETEC toxoid fusion, and ETEC CFA/I/II/IV MEFA), ShigVax (gray box with diagonal lines; *Shigella* MEFA), MecVax (gray box; ETEC toxoid fusion and CFA/I/II/IV MEFA), or PBS (black box). (B) Antibody invasion inhibition from the sera of the mice immunized with ShecVax (white; three proteins), ShigVax (gray; *Shigella* MEFA), or PBS (black) against *Shigella* flexneri 2a, 3a, 6, *S. sonnei*, *S. boydii*, or *S. dysenteriae* bacteria (%; the invaded CFUs of each strain treated with the control sera as 100%). (C) Antibody adherence inhibition from the sera of the mice immunized with ShecVax (white), MecVax (gray), or PBS (black) against ETEC bacteria H10407 (CFA/I), EL392-75 (CS1), ETP05011 (CS2), E116 (CS3), E106 (CS4), UM75688 (CS5), or B7A (CS6), with adherent CFUs from each strain treated with the control sera as 100%. Boxes and bars indicate means and deviations, and ***P < 0.0001.

Combining three polyvalent protein antigens for i.m. immunization did not compromise the antigenicity of individual antigens. Mice i.m. immunized with the three proteins (ShecVax), *Shigella* MEFA protein alone (ShigVax), or two ETEC antigens (toxoid fusion 3xSTa_N12S_–mnLT_R192G/L211A_ and CFA/I/II/IV MEFA; MecVax) developed the same or similar levels of IgG responses to the target antigens. The serum IgG titers to the *Shigella* antigens in the mice i.m. immunized with ShecVax (three proteins) or with *Shigella* MEFA alone (ShigVax) were the same or comparable. Similarly, the IgG titers to the ETEC antigens in the mice immunized with ShecVax (three proteins) or ETEC toxoid fusion 3xSTa_N12S_–mnLT_R192G/L211A_ and CFA/I/II/IV MEFA (MecVax) showed no significant differences (Fig. 4A).

Moreover, mouse serum antibodies derived from the immunization with three polyvalent proteins or individual protein(s) displayed the same level of *in vitro* functions against *Shigella* bacterial invasion, ETEC bacterial adherence, or ETEC STa or LT (CT) toxin enterotoxicity. The sera from the mice immunized with ShecVax or ShigVax equally inhibited *in vitro* invasion of *Shigella* species and serotype strains (Fig. 4B).

Similarly, the sera from the mice immunized with ShecVax or the two ETEC antigen components (MecVax) equivalently inhibited adherence to the target ETEC pathotypes (Fig. 4C). In addition, the sera from these two groups showed no differences when tested for neutralization activities against STa or CT. The intracellular cGMP levels in T-84 cells incubated with STa toxin treated with sera of the mice immunized with MecVax (3xSTa_N12S_–mnLT_R192G/L211A_ and CFA/I/II/IV MEFA) were 4.9 ± 0.62 nM, not different from the cGMP from the treatment with the sera of the mice immunized with ShecVax (3.7 ± 0.33 nM). The intracellular cAMP levels in T-84 cells incubated with CT pretreated with the sera of the mice vaccinated with MecVax or ShecVax were also the same, 5.3 ± 0.39 and 5.0 ± 0.62 nM, respectively.

### ShecVax protects against *Shigella* lethal pulmonary infection

**Fig 5:**
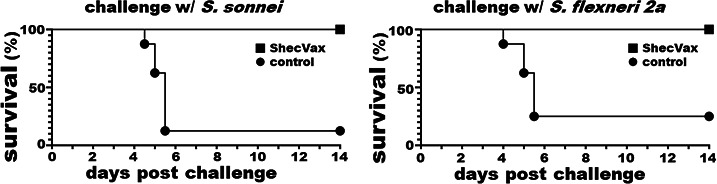
ShecVax protection against *Shigella* lethal pulmonary infections. Mice i.n. immunized with ShecVax (■) or PBS (●), adjuvanted with double-mutant LT (dmLT) (2.5 µg), were infected with 1 × 10^6^ CFUs of *S. sonnei* (*n* = 6) or *S. flexneri* 2a (*n* = 8) in a lethal pulmonary infection model. Mice were monitored for survival up to 14 days post-inoculation. Those who became nonresponsive or lost 20% of body weight over 48 h were euthanized and recorded as dead.

Mice intranasally (i.n.) immunized with ShecVax were protected from lethal pulmonary infection with *S. sonnei* or *S. flexneri* 2a (Fig. 5). After being challenged with *S. sonnei*, all six immunized mice were protected from lethal infection. In contrast, only one control mouse survived. The other five control mice became unresponsive or lost over 20% of their body weight within 48 h. When challenged with *S. flexneri 2*a, all seven immunized mice survived (one mouse was lost before infection, but medical exams showed no sign of infections). In contrast, six control mice became unresponsive or lost more than 20% of body weight in 48 h; only two control mice survived.

IgG responses to all the antigens targeted by ShecVax except for STa (0.5 ± 1.2; log_10_) were detected from the sera (collected at euthanasia, after pulmonary infection) of the immunized mice, but not from the control mice (which were i.n. immunized with PBS and adjuvant double-mutant LT [dmLT]) except for LT (5.0 ± 0.17; due to adjuvant dmLT, a double mutant LT) and mild IgG responses to IpaD (1.4 ± 2.1), IpaB (1.5 ± 2.2), CS1 (1.1 ± 1.6), CS5 (1.2 ± 1.7), or CS6 (0.5 ± 1.3).

IgA responses were not detected in mouse serum samples. Mouse feces and lung tissues were not collected, thus not tested for IgG or IgA responses in this study.

### ShecVax protects against ETEC bacteria colonization in the small intestine

**Fig 6:**
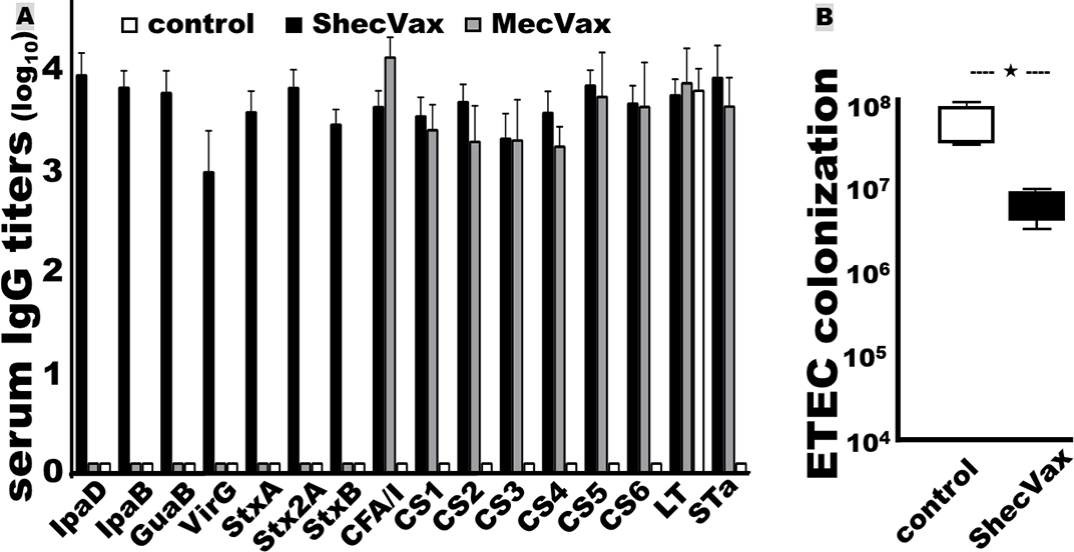
Rabbit serum antigen-specific IgG titers (log_10_) and ShecVax protection against ETEC colonization in rabbit small intestines. (A) Serum IgG titers to *Shigella* IpaD, IpaB, GuaB, VirG, StxA, Stx2A, StxB, ETEC adhesins CFA/I, CS1, CS2, CS3, CS4, CS5, CS6, and ETEC toxins LT and STa from the rabbits (*n* = 4) i.m. immunized with ShecVax (black box), ETEC vaccine MecVax (grey box), or PBS (white box), adjuvanted with 1 µg of dmLT. (B) ShecVax protection against ETEC H10407 bacteria colonization in rabbit small intestines. Rabbits i.m. immunized with ShecVax (black box) or PBS (white box) were intragastrically inoculated with ETEC H10407 (1 × 10^9^ CFUs), then euthanized and necropsied 24 h post-inoculation. An ileum distal segment (10 cm) collected from each rabbit was ground in sterile PBS (1:10), serially diluted, plated on agar plates, and cultured. Bacteria (CFUs; in %) were counted after overnight growth. Boxes and bars indicate means and deviations, and *P < 0.01.

Rabbits i.m. immunized with ShecVax developed IgG antibodies (Fig. 6A). The IgG titers to the ETEC antigens were not different than the titers in the rabbits i.m. immunized with ETEC vaccine candidate MecVax. No antigen-specific IgA responses were detected from rabbit sera or cecum contents.

After oral challenge with ETEC strain H10407 (CFA/I, STa, LT), the immunized rabbits had significantly fewer ETEC bacteria colonized in the small intestine (7.5 ± 3.0 × 10^6^ CFUs per gram of ileum) compared to the control rabbits (6.3 ± 3.7 × 10^7^ *P* < 0.01) (Fig. 6B). The reduction levels of ETEC bacterial intestinal colonization in the rabbits immunized with ShecVax were similar to those vaccinated with MecVax (3.1 ± 1.3 × 10^5^; *P* > 0.10).

### ShecVax passively protects against ETEC toxin-mediated clinical diarrhea

**Fig 7:**
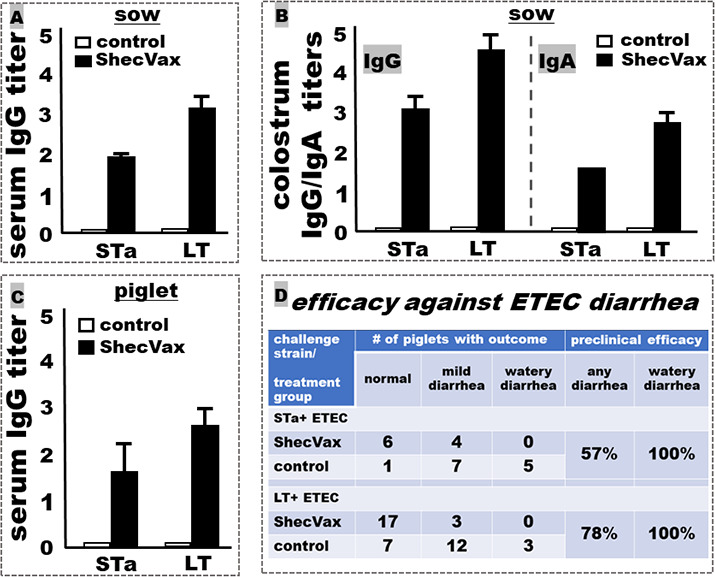
IgG and IgA titers (log_10_) to ETEC toxins (STa, LT) in pigs and ShecVax protection against ETEC toxin-mediated clinical diarrhea. (A) Anti-STa and anti-LT IgG titers in the sera of the pregnant sows i.m. immunized with ShecVax (black box) or PBS (white box). Sows (*n* = 2) were i.m. immunized with ShecVax 6–8 weeks before farrowing, followed by a booster 4 weeks later. Sera were collected at farrowing. (B) Anti-STa and anti-LT IgG and IgA titers in the colostrum samples collected from the sows i.m. immunized with ShecVax (black box) or PBS (white box). (C) Anti-STa and anti-LT IgG titers in the sera of the piglets born to the sows immunized with ShecVax (black box) or PBS (white box). (D) Clinical outcomes in piglets after oral inoculation with an STa+ or LT+ ETEC strain. Suckling piglets born to the immunized mothers or the control mothers were orally inoculated with STa+ ETEC recombinant strain 8651 or LT+ ETEC strain 8025 and recorded for vomiting, lethargy, diarrhea (mild, watery), and dehydration for 24–36 h post-inoculation.

Pregnant sows i.m. immunized with ShecVax developed IgG (serum, colostrum) and IgA (colostrum) to the target antigens (Fig. 7A and B showed IgG and IgA to ETEC toxins STa and LT). Piglets born to the sows i.m. immunized with ShecVax acquired antibodies (Fig. 7C showed serum IgG to STa and LT) and were protected from clinical diarrhea after challenge with an STa+ or LT+ ETEC strain (Fig. 7D). Challenged with an STa-producing recombinant ETEC strain (pig-specific adhesin K88, STa), none of the 10 piglets born to the two immunized mothers developed watery diarrhea, with four piglets having fecal stains at the rear buttocks (considered mild diarrhea). In contrast, 12 of 13 piglets born to two control mothers developed watery diarrhea (100% efficacy against STa-mediated watery diarrhea, 57% against any diarrhea).

Challenged with an LT-positive recombinant ETEC strain (pig-specific adhesin K88, LT), 17 of 20 piglets born to two immunized mothers remained healthy, and only three showed mild diarrhea. In contrast, 15 of 22 piglets born to two control mothers developed diarrhea (100% efficacy against LT-mediated watery diarrhea, 78% against any diarrhea).

## DISCUSSION

This study found that a MEFA-assisted *Shigella* and ETEC combined vaccine candidate (ShecVax) was broadly immunogenic in mice, rabbits, and pigs, and it induced antibodies to the target antigens. The induced antibodies were directed to each epitope incorporated into each MEFA protein immunogen. These antibodies were functional antibodies and protected animals against *Shigella* and ETEC challenges.

To develop MEFA-based polyvalent antigens for a *Shigella* and ETEC combined vaccine, we need to identify the immunodominant or better functional epitopes of target virulence determinants from two heterogeneous groups of bacteria. For ETEC, we targeted seven ETEC adhesins (CFA/I, CS1–CS6) and two toxins (STa, LT) to protect against all strains of ETEC. These seven ETEC adhesins are associated with two-thirds of clinical cases, and the two enterotoxins, alone or together, are produced by all ETEC strains. For *Shigella*, we selected the key virulence proteins that are common to all *Shigella* strains, as well as Shiga toxins, to provide extensive coverage and not be dependent on species or serotypes. By combining these antigens, ShecVax is designed to protect against these two enteric pathogens that cause the highest disease burden in children and travelers.

MEFA-assisted antigens and vaccines, especially combined vaccines, provide a solution to solve the virulence heterogeneity challenge in vaccine development. Virulence heterogeneity is a common challenge in vaccine development. Conventional approaches to overcome virulence heterogeneity include the development of cocktail vaccines or vaccines with conserved antigens. A cocktail vaccine composed of multiple cellular components (live attenuated or killed microorganisms) or acellular antigens (macromolecules including toxoids, viral particles, lipopolysaccharides, or proteins) can broaden protection coverage. However, mixing multiple microorganisms (or molecules) can complicate vaccine formulation and manufacturing (39). A cocktail product contains excessive somatic antigens, especially a mixture of a few whole-cell microorganisms. Some somatic antigens in whole-cell products, particularly bacteria endotoxins, can potentially introduce side effects and risk vaccine safety. Excessive somatic antigens, in addition, can potentially divert hosts from responding to the key virulence determinants and thus reduce or diminish vaccine efficacy. Using a mixture of conserved virulence determinants as vaccine antigens can alternatively overcome the heterogeneity challenge, but identifying them among divergent pathogenic strains, especially those that are antigenically divergent or constantly evolve or resort, will encounter technical difficulties. To provide the same coverage as ShecVax, a cocktail whole-cell vaccine product would be composed of up to seven ETEC strains and six *Shigella* strains, and a cocktail acellular product would contain up to eight ETEC proteins (seven adhesins and one toxoid fusion) and five *Shigella* proteins (IpaB, IpaD, VirG, GuaB, and a Shiga toxoid). Even using conserved virulence antigens, a combined vaccine for *Shigella* and ETEC would carry at least *Shigella* IpaB, IpaD (or an IpaB and IpaD fusion), VirG, and a Shiga toxoid, in addition to an ETEC toxoid fusion, and perhaps a few adhesin subunit proteins. Conventional approaches will, therefore, encounter enormous complexity challenges to achieve an effective *Shigella* and ETEC vaccine.

Different from conventional approaches, the MEFA combines epitope vaccinology and structure vaccinology concepts and integrates multiple antigenic domains or epitopes from heterogeneous virulence factors or strains to create a chimeric polyvalent immunogen. By presenting conserved and functional epitopes of multiple virulence determinants or strains on a backbone immunogen and mimicking epitope native antigenicity (31), MEFA-assisted vaccines overcome the virulence heterogeneity challenge, enabling the development of a broadly immunogenic and cross-protective vaccine. In addition, unlike a mixture of whole-cell microbes or macromolecules, a MEFA-based immunogen consists of only functional epitopes from virulence determinants without somatic antigens. A MEFA-assisted vaccine, being antigenically defined, precisely targets virulence determinants and cross-protects against different pathogenic strains (or diseases), thus improving vaccine immunity, safety, and efficiency.

Data from the current study showed that this *Shigella*/ETEC combined vaccine candidate, ShecVax, is broadly immunogenic and cross-protective against *Shigella* and ETEC infections. Mice i.m. immunized with ShecVax developed IgG antibody responses to all the target virulence factors, and the derived antibodies inhibited invasion from different *Shigella* species and serotypes, prevented the adherence from ETEC strains expressing any of the target adhesin (CFA/I, CS1 to CS6), and neutralized ETEC STa or LT enterotoxicity. Moreover, ShecVax protected mice against *S. sonnei* and *S. flexneri* lethal pulmonary infections, significantly reducing ETEC H10407 bacterial colonization in rabbit small intestines, and passively preventing newly born piglets from ETEC toxin-mediated clinical watery diarrhea.

Additionally, data revealed that the three MEFA-based polyvalent protein antigens are antigenically compatible. Antigen-specific IgG responses from the mice after i.m. immunization with ShecVax (three antigens) were at similar levels to those in the mice immunized with ShigVax (*Shigella* MEFA) or MecVax (two ETEC antigens). Moreover, the *in vitro* antibody functions and *in vivo* preclinical protection data indicated that ShecVax equally protects against *Shigella* bacteria invasion and lethal pulmonary infection as *Shigella* MEFA (ShigVax, a *Shigella* standalone vaccine candidate) does. ShecVax and MecVax (an ETEC standalone vaccine candidate) are equally effective against ETEC bacterial adherence, STa and LT enterotoxicity, ETEC bacteria intestinal colonization, and ETEC toxin-mediated clinical diarrhea. These data support the development of a cross-protective *Shigella*/ETEC combined vaccine. Results from this study perhaps further validate the general application of this MEFA platform for the construction of epitope- and structure-based polyvalent protein antigens and the development of cross-protective vaccines.

Data from this study showed that the combined vaccine induced robust antibody responses to all target virulence factors represented by the epitopes of the MEFA protein antigens. This indicated that the individual epitopes were immunogenic. Moreover, the vaccine-induced antibodies were functional against each virulence factor based on *in vitro* assays, suggesting that individual epitopes on these MEFA immunogens are not compromised or interfered with by each other antigenically and functionally. It may be a concern that integrating multiple foreign epitopes, particularly immunodominant epitopes, into a backbone immunogen by this MEFA platform could result in antigen dominance or antigenic interference. Though we cannot eliminate potential antigenic dominance or interference from some epitopes to the other epitopes on a MEFA immunogen, such risk is preventable and can be minimized. *In silico* prediction of immunodominant epitopes (with online B-cell epitope prediction programs, including BepiPred and IEDB) or empirical identification of functional epitopes (through epitope mapping) results in a panel of candidate epitopes for a virulence factor or strain of interest. When a top-ranked epitope is often selected and integrated into a MEFA backbone immunogen, *in silico* optimization will comparatively examine the antigenicity score and surface area of each epitope on the MEFA versus the native virulence factor (31). Suppose antigenicity is significantly altered or unexpectedly dominant; in that case, this epitope will be placed at a different location on the MEFA backbone or replaced with another epitope from the same virulence factor. Thus, antigenicity dominance or interference among the epitopes on a MEFA immunogen can be reduced or prevented. Preventing interference of epitope function, however, can only be confirmed empirically. Antibodies derived from ShecVax showed *in vitro* protection against each virulence factor, suggesting that the representing epitopes on the three MEFA protein immunogens retain their functions. Future function studies targeting each individual epitope can determine epitope functional interference when it is on the MEFA immunogens with other epitopes or as the only epitope.

One limitation of the study is the use of only a few challenge strains in ShecVax protection studies. The *Shigella* pulmonary infection experiment used two strains, *S. sonnei* and *S. flexneri* 2a. Future challenge studies including *S. flexneri* serotypes 3a and 6, and species *S. boydii* and *S. dysenteriae* can better assess ShecVax cross-protection against *Shigella* infections. Similarly, only the most virulent ETEC strain, H10407, was included in the rabbit colonization study to evaluate ShexVax efficacy against ETEC colonization in the small intestines. Future studies to have additional ETEC strains expressing the other six targeted adhesins can better evaluate ShecVax protection against ETEC intestinal colonization.

The current study focused primarily on IgG responses, as antigen-specific IgA responses were not detected in the mice or rabbits i.m. immunized with ShecVax. That is similar to what we reported previously, i.e., mice i.m. immunized with the *Shigella* MEFA protein or the two ETEC polyvalent proteins of MecVax ETEC, adjuvant with 0.1 µg, had robust antigen-specific IgG, but no IgA, detected from serum samples (32, 37, 40). On the other hand, we noticed that pregnant sows developed IgG and IgA in colostrum after i.m. immunization with ShecVax (with 5 µg of dmLT; Fig. 7), the same as the pregnant sows i.m. immunized with ETEC vaccine candidate MecVax in a previous study (37). Interestingly, pregnant rabbits developed IgG and IgA in milk as well after i.m. immunization with a cholera MEFA protein (41). It is unclear to us momentarily whether the amount of dmLT adjuvant or/and the maternal status play a role in eliciting IgA responses. However, it was reported that mice i.m. immunized with ETEC CS6 adhesin subunit protein, adjuvanted with 0.5 µg of dmLT, developed serum IgG and IgA antibodies in a dose-dependent manner (42). While IgA, particularly local mucosal IgA antibodies, are believed to play a more important role in protection against enteric infections, including *Shigellosis* and ETEC diarrhea, we have shown that rabbits i.m. immunized with MecVax or a cholera MEFA protein developed IgG (no detectable IgA) antibodies and were protected against ETEC or vibrio intestinal colonization (40, 41, 43). Future studies with a high dose of dmLT adjuvant should be informative if ShecVax can also induce antigen-specific serum IgA antibodies in i.m. immunized mice. It will also be interesting if rabbits can develop IgA responses and are better protected against ETEC or vibrio intestinal colonization after i.m. immunization with MecVax or ShecVax with a different adjuvant.

Additionally, we used only B-cell epitopes to construct the polyvalent ETEC or *Shigella* MEFA immunogens and measured only antibody responses from the animals immunized with ShecVax in the current study. While the ETEC toxoid fusion 3xSTa_N12S_–mnLT_R192G/L211A_ retains the T-cell epitopes of the LT A and B subunit peptides, and the *Shigella* MEFA protein may contain IpaD backbone T-cell epitopes, we did not examine T-cell responses or lasting immunity or protection from ShecVax or any of the three polyvalent antigens. Future studies to measure vaccine T-cell responses can help us better characterize this vaccine immunogenicity and perhaps correlate to protection.

An effective combined vaccine for *Shigella* and ETEC could significantly reduce the diarrheal disease burden since *Shigella* and ETEC are the two top bacterial causes of children’s diarrhea (44, 45), and ETEC is the most common cause of travelers’ diarrhea (46). While we are currently developing protein-based standalone vaccines for *Shigella* (ShigVax) or ETEC (MecVax)(32, 34, 37, 40), we prefer a combined vaccine since ETEC and *Shigella* infect the same populations, young children in LMICs and international travelers. Data from this study are very encouraging as they suggest that the antigens of the two standalone vaccines can be combined. A combined vaccine would be preferred in routine vaccination programs for children or travelers, considering the increasing number of vaccines scheduled for each group. However, the efficacy of this combined vaccine candidate against clinical shigellosis and ETEC diarrhea will need to be evaluated in future clinical trials.

## MATERIALS AND METHODS

### Bacteria and plasmids

Plasmids and *Shigella* or ETEC bacteria used in this study are listed in Table 1. *S. flexneri* 2a, 3a, 6, *S. boydii*, and *S. dysenteriae* used in antibody invasion inhibition assays and mouse lethal pulmonary challenge studies were provided by Dr. EM Barry (University of Maryland) and the US National Institute of Health Biodefense and Emerging Infections Research Resources Repository (BEI). ETEC field isolates expressing CS2/CS3, CS3, CS4/CS6, or CS6 for antibody adherence inhibition assays were provided by Dr. JM Fleckenstein (University of Washington at St. Louis), Dr. AM Svennerholm (University of Gothenburg), and PATH. The other *Shigella* (*S. sonnei*) and ETEC (CFA/I, CS1/CS3, SC5/CS6) strains were from Sack laboratory and Zhang laboratory. Plasmids carrying ShecVax vaccine antigen genes and *Shigella* or ETEC virulence factors (for recombinant proteins as antibody titration ELISA coating antigens) were generated in the Zhang laboratory.

**TABLE 1:** *Shigella* and enterotoxigenic *E. coli* (ETEC) strains and plasmids used in the study

Strain/plasmid	Relevant properties	Source
*Strain* *Shigella*		
9856	*Shigella sonnei* 53G	Johns Hopkins University
9905	*Shigella flexneri* 2a 2547T	University of Maryland
9761	*Shigella flexneri* 3a J17A	University of Maryland
9757	*Shigella flexneri* 6 CCHO60	University of Maryland
9857	*Shigella boydii* serotype NCTC 12985 (NR-521; ATCC 8700)	BEI
9786	*Shigella dysenteriae* type 1 Newcastle 1934 (NR520; ATCC 13313)	BEI
*E. coli*		
H10407	O78:H11; CFA/I, STa, LT	Johns Hopkins University
EL392-75	CS1, CS3, STa, LT	Johns Hopkins University
ETP05011	CS2, CS3, STa, LT	University of Washington at St. Louis
E116	CS3, STa, LT	University of Gothenburg
E106	CS4, CS6, STa, LT	University of Gothenburg
UM75688	CS5, CS6, STa, LT	Johns Hopkins University
B7A	CS6, STa, LT	PATH
8651	K88, STa	(47)
8025	K88, LT	(48)
*Plasmid*		
p9475	“*Shigella* MEFA + pET28a” in BL21	(32)
p9471	“3xSTa_N12S_–mnLT_R192G/L211A_ + pET28a” in BL21	(38)
p9472	“CFA/I/II/IV MEFA + pET28a” in BL21	(38)
p9775	“Stx1A + pET28α” in BL21	(32)
p9777	“Stx2A + pET28α” in BL21	(32)
p9845	“Stx1B + pET28α” in BL21	(32)
p9847	“CsaB-VirG-epitope (^193^SDSDGGNGGD^202^) + pET28α” in BL21	(32)
p9756	“GuaB_(1-300)_ + pET28” in BL21	(32)
p9562	“CfaB + pET28a” in BL21	(40)
p9722	“CooA + pET28a” in BL21	(40)
p9723	“CotA + pET28a” in BL21	(40)
p9724	“CstH + pET28a” in BL21	(40)
p9766	“CsaB + pET28a” in BL21	(40)
p9658	“CsfA + pET28a” in BL21	(40)
p9558	“CssA + pET28a” in BL21	(40)

### Mouse i.m. immunization with ShecVax, antibody titration, and antibody functional assays

*Mouse immunization*: Eight-week-old BALB/c female mice, 12 mice per group, were i.m. immunized with ShecVax composed of 20 µg of *Shigella* MEFA protein, 20 µg of CFA/I/II/IV MEFA, and 20 µg of toxoid fusion 3xSTa_N12S_–mnLT_R192G/L211A_, in a total volume of 40 µL. Adjuvant dmLT (dmLT, LT_R192G/L211A_; 0.1 µg in 1 µL) was included in the immunization group. A group of mice i.m. injected with PBS (40 µL) was used as the control. Two boosters followed the primary vaccination at an interval of 2 weeks. Sera were collected from each mouse before the primer and 2 weeks after the final booster and stored at −80°C until use. Using 12 mice per group is for collecting sufficient samples for antibody titration against seven *Shigella* and nine ETEC virulence factor antigens, and antibody functional assays against six *Shigella* spp. and serotypes, seven ETEC strains, and two ETEC toxins.

Two additional groups of mice were included to examine the antigenic compatibility for the three antigens of ShecVax. One group was i.m. immunized with 20 µg of *Shigella* MEFA protein (*Shigella* vaccine ShigVax), and the other was i.m. immunized with 20 µg of CFA/I/II/IV MEFA and 20 µg of toxoid fusion 3xSTa_N12S_–mnLT_R192G/L211A_ (ETEC vaccine MecVax).

*Antigen-specific antibody titration*: Sera collected from each mouse before the primary and 2 weeks after the 2nd booster were examined for IgG and IgA antibodies specific to *Shigella* virulence factors (IpaB, IpaD, VirG, GuaB, StxA, Stx2A, StxB) and ETEC virulence factors (CFA/I, CS1, CS2, CS3, CS4, CS5, CS6, STa, LT) in ELISAs, with each virulence factor recombinant proteins, STa–ovalbumin conjugates, or LT homolog cholera toxin (CT; Sigma) as the respective coating antigen as we previously described (32, 37). Briefly, each recombinant protein (100 ng per well) or STa–ovalbumin conjugate (10 ng per well) was coated to a 96-well 2HB plate (Thermo Fisher Scientific; Waltham, MA) at 4°C overnight. Wells were blocked with 10% non-fat milk, then incubated with twofold serum serial dilutions at 37°C for 1 h. Wells were washed and incubated with HRP-conjugated goat-anti-mouse IgG or IgA (1:5,000; Bethy Laboratories, Montgomery, TX) at 37°C for 1 h, then washed and incubated with 3,3′5,5′-tetramethylbenzidine microwell peroxidase substrate system 2C (Thermo Fisher Scientific). Optical density (OD) was read at 650 nm wavelength after 25 min at room temperature. The highest dilutions that gave an OD_650_ above 0.3 after subtraction of background readings were converted to antibody titers and expressed in log_10_.

*Mouse serum antibody functional assays*: Mouse sera were examined for antibody functions against *Shigella* bacteria invasion, ETEC bacteria adherence, and ETEC toxin enterotoxicity, as we described previously (32, 37).

To examine antibody function against *Shigella* bacteria invasion, *S. flexneri* 2a, 3a, 6, *S. sonnei*, *S. boydii*, and *S. dysenteriae* (~2 × 10^7^ bacteria; in 30 µL), after incubation with sera (30 µL heat inactivated, pooled) from the mice immunized with ShecVax, *Shigella* MEFA (ShigVax), or PBS on a shaker (50 rpm) at room temperature for 25 min, were added to confluent HeLa cells (~2.5 × 10^5^; ATCC, CCL-2) in a 24-well plate and cultured at 37°C for 2 h, then treated with gentamicin at 37°C for 2 h (6 h for *S. flexneri* 6) to eliminate extracellular bacteria. Cells were lysed to release invaded bacteria; lysates were serially diluted and plated on agar plates. Invaded *Shigella* bacteria (CFUs) were counted after overnight growth and converted to percentages referred to the CFUs of the bacteria treated with control mouse sera (as 100%).

For antibody function assays against ETEC bacteria adherence, ETEC field strains expressing CFA/I, CS1/CS3, CS2/CS3, CS3, CS4/CS6, CS5/CS6, or CS6 (~3.5 × 10^6^ CFUs), after incubation with sera (15 µL heat inactivated) from the mice immunized with ShecVax, MecVax (two ETEC antigens), or PBS, were added to confluent Caco-2 cells (~7 × 10^5^; ATCC, HTB-37) and incubated at 37°C for 1 h. After washing to remove non-adherence bacteria, cells were lysed. Lysates were serially diluted and plated on agar plates. Adherent ETEC bacteria (CFUs) were counted after overnight growth and converted to percentages referred to the CFUs of the bacteria treated with control mouse sera (as 100%).

To examine antibody neutralization activities against ETEC STa and LT enterotoxicity, STa, CT (LT homolog), T-84 cells (CCL-248; ATCC, Manassas, Virginia, USA), and a cyclic AMP kit or a cGMP EIA kit (Enzo Life Sciences, Farmingdale, NY, USA) were used. Toxin STa (2 ng) or CT (10 ng) was first incubated with 30 µL of heat-inactivated sera of the mice immunized with ShecVax, MecVax (two ETEC antigens), or PBS at room temperature for 30 min, and then added to confluent T-84 cells in a 24-well plate. After incubation for 1 h (for cGMP) or 3 h (for cAMP), cells were rinsed with PBS to remove extracellular cGMP or cAMP and then lysed to release intracellular cGMP or cAMP. Cell lysates were collected and measured for intracellular cGMP or cAMP concentrations (nM, pico moles per ml) following the manufacturer’s protocol (Enzo Life Sciences).

### Mouse intranasal immunization with ShecVax and lethal pulmonary infection with *S. sonnei* and *S. flexneri*

To evaluate ShecVax preclinical efficacy against *Shigella* infections, we intranasally (i.n.) immunized mice with ShecVax and infected mice with *S. sonnei* or *S. flexneri* 2a in a lethal pulmonary challenge model as we described (32). Eight-week-old BALB/c female mice were sedated with isoflurane and i.n. immunized with ShecVax (20 µg of *Shigella* MEFA protein, 10 µg of CFA/I/II/IV MEFA protein, and 10 µg of toxoid fusion 3xSTa_N12S_–mnLT_R192G/L211A_; in 22.5 µL) or PBS as control, by pipetting droplets into nasal cavities, one prime and two boosters at a 2-week interval, adjuvanted with 2.5 µg of dmLT (in 2.5 µL). Two weeks after the second booster, mice were challenged intranasally with 1 × 10^6^ CFUs (in 1 mL) of *S. sonnei* 53G or *S. flexneri* 2 a bacteria. Challenged mice were closely monitored and weighed daily for up to 2 weeks; those who were not responsive or lost 20% of body weight within 48 h were terminated early and recorded as dead. Sera were collected 2 weeks post-challenge (the survived mice) or at euthanasia (those terminated early) and examined for antibody responses. Efficacy against *Shigella* lethal pulmonary infection was calculated as “(percent death of control mice − percent death of immunized mice)/(percent death of control group).”

### Rabbit i.m. immunization with ShecVax and challenge with ETEC H10407

To assess ShecVax preclinical efficacy against ETEC bacteria intestinal colonization, we i.m. immunized rabbits with ShecVax and challenged the rabbits with ETEC bacteria H10407 (CFA/I, STa, LT), as described previously (34, 40). New Zealand White rabbits (Charles River Laboratories, Wilmington, MA), 1.2–1.5 kg, four per group, were i.m. immunized with ShecVax (250 µg of *Shigella* MEFA protein, 250 µg of CFA/I/II/IV MEFA protein, and 250 µg of toxoid fusion 3xSTa_N12S_–mnLT_R192G/L211A_; in 375 µL) or PBS as control, one prime and two boosters in a 2-week interval, adjuvanted with 1 µg of dmLT. Another group of rabbits i.m. immunized with 250 µg of CFA/I/II/IV MEFA protein and 250 µg of toxoid fusion 3xSTa_N12S_–mnLT_R192G/L211A_ (in 250 µL) was included. Two weeks after the second booster, rabbits were first administered with an antacid drug (famotidine), sedated (dexmedetomidine), anesthetized (isoflurane), inoculated with sodium bicarbonate, then challenged with 1 × 10^11^ CFU ETEC H10407 bacteria (in 1 mL), and followed with sodium bicarbonate. Rabbits were sacrificed 24 h post-challenge. At necropsy, the distal ileum segment (10 cm) was collected, ground (1 g in 9 mL of PBS), serially diluted, and plated on MacConkey agar plates. Bacteria were counted after overnight growth at 37°C and converted to percentages referred to the CFUs of the bacteria from the control rabbits (as 100%).

Rabbit serum and cecum contents were collected from each rabbit at necropsy. Cecum contents were suspended in fecal reconstitution buffer (10 mM Tris, 100 mM NaCl, 0.05% Tween-20, and 5 mM sodium azide, pH 7.4); supernatants were collected and stored at −20°C. Rabbit sera were titrated for IgG and IgA responses to the target antigens, and rabbit cecum content supernatant was examined for antigen-specific IgA responses in ELISAs.

### Pregnant sow i.m. immunization with ShecVax and born piglet challenge with an STa+ or LT+ ETEC strain

A pig passive protection model was used to evaluate ShecVax protection against ETEC toxin-mediated clinical diarrhea. This model immunizes pregnant sows with the combined vaccine candidate and then challenges newly born piglets with a pig-specific ETEC recombinant strain that produces STa or LT toxin. As we described previously (37, 49–51), pregnant sows, four per group, were i.m. immunized with ShecVax (250 µg of *Shigella* MEFA protein, 250 µg of CFA/I/II/IV MEFA protein, and 250 µg of toxoid fusion 3xSTa_N12S_–mnLT_R192G/L211A_, in 750 µL; adjuvanted with 5 µg of dmLT), or PBS (without dmLT adjuvant) as the control, 4–6 weeks before the expected farrowing date, followed with a booster 2 weeks later. Sera and colostrum samples were collected from each sow during farrowing.

After suckling for 3 days, piglets born to two immunized mothers and two control mothers were inoculated orally with 5 × 10^9^ CFU STa-producing ETEC recombinant strain 8651 (K88, STa) (47), and piglets born to the other two immunized mothers and two control mothers were challenged with 5 × 10^9^ CFU LT-producing strain 8025 (K88, LT) (48). Piglets were monitored and recorded for clinical diarrhea every 2 to 4 hs in 24 hpost-inoculation. Vaccine efficacy was measured “(percent of diarrhea in piglets born to the control mothers − percent of diarrhea in piglets born to the immunized mothers)/(percent of diarrhea in piglets born to the control mothers)” and presented in percentage.

### Statistical analyses

Differences between the immunized and control groups were analyzed with the program one-way analysis of variance (ANOVA), with a *post hoc* Tukey’s test used to calculate *P* values between the two groups. A *P*-value less than 0.05 was considered significant.

## References

[1] Collaborators GDD. 2018. Estimates of the global, regional, and national morbidity, mortality, and aetiologies of diarrhoea in 195 countries: a systematic analysis for the Global Burden of Disease Study 2016. Lancet Infect Dis 18:1211–1228. 30243583 10.1016/S1473-3099(18)30362-1 PMC6202444

[2] Olson S, Hall A, Riddle MS, Porter CK. 2019. Travelers’ diarrhea: update on the incidence, etiology and risk in military and similar populations - 1990-2005 versus 2005-2015, does a decade make a difference? Trop Dis Travel Med Vaccines 5:1. doi:10.1186/s40794-018-0077-1 30675367 PMC6332902

[3] Walker R, Dull P. 2017. Combination vaccine strategies to prevent enteric infections. Vaccine (Auckl) 35:6790–6792. doi:10.1016/j.vaccine.2017.06.076 28705515

[4] Kotloff KL, Platts-Mills JA, Nasrin D, Roose A, Blackwelder WC, Levine MM. 2017. Global burden of diarrheal diseases among children in developing countries: incidence, etiology, and insights from new molecular diagnostic techniques. Vaccine (Auckl) 35:6783–6789. doi:10.1016/j.vaccine.2017.07.036 28765005

[5] Trust W. 2018. Vaccines to tackle drug resistant infections: an evaluation of R&D opportunitie 2018. Available from: https://vaccinesforamr.org/wp-content/uploads/2018/09/Vaccines_for_AMR.pdf

[6] Hosangadi D, Smith PG, Kaslow DC, Giersing BK, Who E. 2019. WHO consultation on ETEC and Shigella burden of disease, Geneva, 6-7th April 2017: meeting report. Vaccine (Auckl) 37:7381–7390. doi:10.1016/j.vaccine.2017.10.011 29352598

[7] Venkatesan MM, Van de Verg LL. 2015. Combination vaccines against diarrheal diseases. Hum Vaccin Immunother 11:1434–1448. doi:10.4161/21645515.2014.986984 25891647 PMC4517455

[8] WHO. 2006. Future directions for research on enterotoxigenic Escherichia coli vaccines for developing countries. Wkly Epidemiol Rec 81:97–107. 16671213

[9] Tribble DR. 2017. Resistant pathogens as causes of traveller’s diarrhea globally and impact(s) on treatment failure and recommendations. J Travel Med 24:S6–S12. doi:10.1093/jtm/taw090 28520997 PMC5731445

[10] Lääveri T, Vilkman K, Pakkanen S, Kirveskari J, Kantele A. 2018. Despite antibiotic treatment of travellers’ diarrhoea, pathogens are found in stools from half of travellers at return. Travel Med Infect Dis 23:49–55. doi:10.1016/j.tmaid.2018.04.003 29702254

[11] Humphries RM, Schuetz AN. 2015. Antimicrobial susceptibility testing of bacteria that cause gastroenteritis. Clin Lab Med 35:313–331. doi:10.1016/j.cll.2015.02.005 26004645

[12] Nunes MRCM, Magalhães PP, Penna FJ, Nunes JMM, Mendes EN. 2012. Diarrhea associated with Shigella in children and susceptibility to antimicrobials. J Pediatr (Rio J) 88:125–128. doi:10.2223/JPED.2131 22089139

[13] Ouyang-Latimer J, Jafri S, VanTassel A, Jiang ZD, Gurleen K, Rodriguez S, Nandy RK, Ramamurthy T, Chatterjee S, McKenzie R, Steffen R, DuPont HL. 2011. In vitro antimicrobial susceptibility of bacterial enteropathogens isolated from international travelers to Mexico, Guatemala, and India from 2006 to 2008. Antimicrob Agents Chemother 55:874–878. doi:10.1128/AAC.00739-10 21115800 PMC3028774

[14] Streit JM, Jones RN, Toleman MA, Stratchounski LS, Fritsche TR. 2006. Prevalence and antimicrobial susceptibility patterns among gastroenteritis-causing pathogens recovered in Europe and Latin America and Salmonella isolates recovered from bloodstream infections in North America and Latin America: report from the SENTRY Antimicrobial Surveillance Program (2003). Int J Antimicrob Agents 27:367–375. doi:10.1016/j.ijantimicag.2005.12.004 16647842

[15] von Seidlein L, Kim DR, Ali M, Lee H, Wang X, Thiem VD, Canh DG, Chaicumpa W, Agtini MD, Hossain A, Bhutta ZA, Mason C, Sethabutr O, Talukder K, Nair GB, Deen JL, Kotloff K, Clemens J. 2006. A multicentre study of Shigella diarrhoea in six asian countries: disease burden, clinical manifestations, and microbiology. PLoS Med 3:e353. doi:10.1371/journal.pmed.0030353 16968124 PMC1564174

[16] Gomi H, Jiang Z-D, Adachi JA, Ashley D, Lowe B, Verenkar MP, Steffen R, DuPont HL. 2001. In vitro antimicrobial susceptibility testing of bacterial enteropathogens causing traveler’s diarrhea in four geographic regions. Antimicrob Agents Chemother 45:212–216. doi:10.1128/AAC.45.1.212-216.2001 11120968 PMC90263

[17] PATH. 2011. The case for investtment in enterotoxigenic Escherichia coli vaccines. PATH, Seattle, WA.

[18] Qadri F, Bhuiyan TR, Sack DA, Svennerholm AM. 2013. Immune responses and protection in children in developing countries induced by oral vaccines. Vaccine (Auckl) 31:452–460. doi:10.1016/j.vaccine.2012.11.012 23153448

[19] Sack DA. 2019. Enhancing immune responses to oral vaccines: still an enigma. Lancet Infect Dis 19:122–123. doi:10.1016/S1473-3099(18)30662-5 30722995

[20] Svennerholm A-M, Tobias J. 2008. Vaccines against enterotoxigenic Escherichia coli. Expert Rev Vaccines 7:795–804. doi:10.1586/14760584.7.6.795 18665777

[21] Zhang W, Sack DA. 2012. Progress and hurdles in the development of vaccines against enterotoxigenic Escherichia coli in humans. Expert Rev Vaccines 11:677–694. doi:10.1586/erv.12.37 22873126

[22] Walker RI. 2015. An assessment of enterotoxigenic Escherichia coli and Shigella vaccine candidates for infants and children. Vaccine (Auckl) 33:954–965. doi:10.1016/j.vaccine.2014.11.049 25482842

[23] Zhang W, Sack DA. 2015. Current progress in developing subunit vaccines against enterotoxigenic Escherichia coli-associated diarrhea. Clin Vaccine Immunol 22:983–991. doi:10.1128/CVI.00224-15 26135975 PMC4550667

[24] Mani S, Wierzba T, Walker RI. 2016. Status of vaccine research and development for Shigella. Vaccine (Auckl) 34:2887–2894. doi:10.1016/j.vaccine.2016.02.075 26979135

[25] Kotloff KL, Simon JK, Pasetti MF, Sztein MB, Wooden SL, Livio S, Nataro JP, Blackwelder WC, Barry EM, Picking W, Levine MM. 2007. Safety and immunogenicity of CVD 1208S, a live, oral DeltaguaBA deltasen deltaset Shigella flexneri 2a vaccine grown on animal-free media. Hum Vaccin 3:268–275. doi:10.4161/hv.4746 17938573

[26] Qadri F, Svennerholm A-M, Faruque ASG, Sack RB. 2005. Enterotoxigenic Escherichia coli in developing countries: epidemiology, microbiology, clinical features, treatment, and prevention. Clin Microbiol Rev 18:465–483. doi:10.1128/CMR.18.3.465-483.2005 16020685 PMC1195967

[27] Svennerholm AM, Lundgren A. 2012. Recent progress toward an enterotoxigenic Escherichia coli vaccine. Expert Rev Vaccines 11:495–507. doi:10.1586/erv.12.12 22551034

[28] Isidean SD, Riddle MS, Savarino SJ, Porter CK. 2011. A systematic review of ETEC epidemiology focusing on colonization factor and toxin expression. Vaccine (Auckl) 29:6167–6178. doi:10.1016/j.vaccine.2011.06.084 21723899

[29] Livio S, Strockbine NA, Panchalingam S, Tennant SM, Barry EM, Marohn ME, Antonio M, Hossain A, Mandomando I, Ochieng JB, et al.. 2014. Shigella isolates from the global enteric multicenter study inform vaccine development. Clin Infect Dis 59:933–941. doi:10.1093/cid/ciu468 24958238 PMC4166982

[30] Levine MM, Kotloff KL, Barry EM, Pasetti MF, Sztein MB. 2007. Clinical trials of Shigella vaccines: two steps forward and one step back on a long, hard road. Nat Rev Microbiol 5:540–553. doi:10.1038/nrmicro1662 17558427 PMC3771495

[31] Zhang W, Li S, Lee K. 2022. Multiepitope fusion antigen: MEFA, an epitope- and structure-based vaccinology platform for multivalent vaccine development. Methods Molecul Biol:151–169. doi:10.1007/978-1-0716-1900-1_10 PMC10294517 34784037

[32] Li S, Anvari S, Ptacek G, Upadhyay I, Kaminski RW, Sack DA, Zhang W. 2023. A broadly immunogenic polyvalent Shigella multiepitope fusion antigen protein protects against Shigella sonnei and Shigella flexneri lethal pulmonary challenges in mice. Infect Immun 91:e0031623. doi:10.1128/iai.00316-23 37795982 PMC10652900

[33] Ruan X, Knudsen DE, Wollenberg KM, Sack DA, Zhang W. 2014. Multiepitope fusion antigen induces broadly protective antibodies that prevent adherence of Escherichia coli strains expressing colonization factor antigen I (CFA/I), CFA/II, and CFA/IV. Clin Vaccine Immunol 21:243–249. doi:10.1128/CVI.00652-13 24351757 PMC3910947

[34] Upadhyay I, Lauder KL, Li S, Ptacek G, Zhang W. 2022. Intramuscularly administered enterotoxigenic Escherichia coli (ETEC) vaccine candidate MecVax prevented H10407 intestinal colonization in an adult rabbit colonization model. Microbiol Spectr 10:e0147322. doi:10.1128/spectrum.01473-22 35762781 PMC9431210

[35] Upadhyay I, Parvej SMD, Shen Y, Li S, Lauder KL, Zhang C, Zhang W. 2023. Protein-based vaccine candidate MecVax broadly protects against enterotoxigenic Escherichia coli intestinal colonization in a rabbit model. Infect Immun. doi:10.1128/iai.00272-23:e0027223 PMC10652908 37874163

[36] Ruan X, Robertson DC, Nataro JP, Clements JD, Zhang W, The STa Toxoid Vaccine Consortium Group. 2014. Characterization of heat-stable (STa) toxoids of enterotoxigenic Escherichia coli fused to double mutant heat-labile toxin peptide in inducing neutralizing anti-STa antibodies. Infect Immun 82:1823–1832. doi:10.1128/IAI.01394-13 24549325 PMC3993458

[37] Seo H, Garcia C, Ruan X, Duan Q, Sack DA, Zhang W. 2021. Preclinical characterization of immunogenicity and efficacy against diarrhea from MecVax, a multivalent enterotoxigenic E. coli vaccine candidate. Infect Immun 89:e0010621. doi:10.1128/IAI.00106-21 33875477 PMC8208516

[38] Duan Q, Lu T, Garcia C, Yanez C, Nandre RM, Sack DA, Zhang W. 2018. Co-administered tag-less toxoid fusion 3xSTaN12S-m_nLTR_192G/_L211A and C_FA/I/II/IV MEFA (multiepitope fusion antigen) induce neutralizing antibodies to 7 adhesins. Front Microbiol 9:e1198. doi:10.3389/fmicb.2018.01198 PMC5996201 29922268

[39] Schlingmann B, Castiglia KR, Stobart CC, Moore ML. 2018. Polyvalent vaccines: high-maintenance heroes. PLoS Pathog 14:e1006904. doi:10.1371/journal.ppat.1006904 29621336 PMC5886581

[40] Upadhyay I, Parvej SMD, Shen Y, Li S, Lauder KL, Zhang C, Zhang W. 2023. Protein-based vaccine candidate MecVax broadly protects against enterotoxigenic Escherichia coli intestinal colonization in a rabbit model. Infect Immun 91:e0027223. doi:10.1128/iai.00272-23 37874163 PMC10652908

[41] Upadhyay I, Li S, Ptacek G, Seo H, Sack DA, Zhang W. 2022. A polyvalent multiepitope protein cross-protects against Vibrio cholerae infection in rabbit colonization and passive protection models. Proc Natl Acad Sci USA 119:e2202938119. doi:10.1073/pnas.2202938119 36469767 PMC9897427

[42] Lee T, Gutiérrez RL, Maciel M, Poole S, Testa KJ, Trop S, Duplessis C, Lane A, Riddle MS, Hamer M, Alcala A, Prouty M, Maier N, Erdem R, Louis Bourgeois A, Porter CK. 2021. Safety and immunogenicity of intramuscularly administered CS6 subunit vaccine with a modified heat-labile enterotoxin from enterotoxigenic Escherichia coli. Vaccine (Auckl) 39:5548–5556. doi:10.1016/j.vaccine.2021.08.032 PMC8461560 34419306

[43] Jones RM, Seo H, Zhang W, Sack DA. 2022. A multi-epitope fusion antigen candidate vaccine for enterotoxigenic Escherichia coli is protective against strain B7A colonization in a rabbit model. PLoS Negl Trop Dis 16:e0010177. doi:10.1371/journal.pntd.0010177 35139116 PMC8863229

[44] Kotloff KL, Nataro JP, Blackwelder WC, Nasrin D, Farag TH, Panchalingam S, Wu Y, Sow SO, Sur D, Breiman RF, et al.. 2013. Burden and aetiology of diarrhoeal disease in infants and young children in developing countries (the Global Enteric Multicenter Study, GEMS): a prospective, case-control study. Lancet 382:209–222. doi:10.1016/S0140-6736(13)60844-2 23680352

[45] Platts-Mills JA, Babji S, Bodhidatta L, Gratz J, Haque R, Havt A, McCormick BJ, McGrath M, Olortegui MP, Samie A, et al.. 2015. Pathogen-specific burdens of community diarrhoea in developing countries: a multisite birth cohort study (MAL-ED). Lancet Glob Health 3:e564–75. doi:10.1016/S2214-109X(15)00151-5 26202075 PMC7328884

[46] Jiang ZD, DuPont HL. 2017. Etiology of travellers’ diarrhea. J Travel Med 24:S13–S16. doi:10.1093/jtm/tax003 28521001

[47] Zhang W, Robertson DC, Zhang C, Bai W, Zhao M, Francis DH. 2008. Escherichia coli constructs expressing human or porcine enterotoxins induce identical diarrheal diseases in a piglet infection model. Appl Environ Microbiol 74:5832–5837. doi:10.1128/AEM.00893-08 18658289 PMC2547035

[48] Zhang W, Berberov EM, Freeling J, He D, Moxley RA, Francis DH. 2006. Significance of heat-stable and heat-labile enterotoxins in porcine colibacillosis in an additive model for pathogenicity studies. Infect Immun 74:3107–3114. doi:10.1128/IAI.01338-05 16714538 PMC1479275

[49] Nandre R, Ruan X, Lu T, Duan Q, Sack D, Zhang W. 2018. Enterotoxigenic Escherichia coli adhesin-toxoid multiepitope fusion antigen CFA/I/II/IV-3xSTaN12S-mnLTG192G/L211A-derived antibodies inhibit adherence of seven adhesins, neutralize enterotoxicity of LT and STa toxins, and protect piglets against diarrhea. Infect Immun 86:e00550-17. doi:10.1128/IAI.00550-17 29263112 PMC5820944

[50] Nandre RM, Duan Q, Wang Y, Zhang W. 2017. Passive antibodies derived from intramuscularly immunized toxoid fusion 3xSTa_N12S_-dmLT protect against STa+ enterotoxigenic Escherichia coli (ETEC) diarrhea in a pig model. Vaccine (Auckl) 35:552–556. doi:10.1016/j.vaccine.2016.12.021 PMC5261774 28017433

[51] Zhang W, Zhang C, Francis DH, Fang Y, Knudsen D, Nataro JP, Robertson DC. 2010. Genetic fusions of heat-labile (LT) and heat-stable (ST) toxoids of porcine enterotoxigenic Escherichia coli elicit neutralizing anti-LT and anti-STa antibodies. Infect Immun 78:316–325. doi:10.1128/IAI.00497-09 19858307 PMC2798211

